# Circadian Genes, the Stress Axis, and Alcoholism

**DOI:** 10.35946/arcr.v34.3.12

**Published:** 2012

**Authors:** Dipak K. Sarkar

**Affiliations:** **Dipak K. Sarkar, Ph.D., D.Phil.,***is the director of and a professor in the Endocrinology Program and Department of Animal Sciences, Rutgers, The State University of New Jersey, New Brunswick, New Jersey.*

**Keywords:** Alcohol consumption, alcohol use, abuse and dependence, alcohol and other drug use pattern, genetics, genetic factors, circadian system, clock genes, stress, stress response, biological adaptation to stress, neurobiology, hypothalamic– pituitary–adrenal axis

## Abstract

The body’s internal system to control the daily rhythm of the body’s functions (i.e., the circadian system), the body’s stress response, and the body’s neurobiology are highly interconnected. Thus, the rhythm of the circadian system impacts alcohol use patterns; at the same time, alcohol drinking also can alter circadian functions. The sensitivity of the circadian system to alcohol may result from alcohol’s effects on the expression of several of the clock genes that regulate circadian function. The stress response system involves the hypothalamus and pituitary gland in the brain and the adrenal glands, as well as the hormones they secrete, including corticotrophin-releasing hormone, adrenocorticotrophic hormone, and glucocorticoids. It is controlled by brain-signaling molecules, including endogenous opioids such as β-endorphin. Alcohol consumption influences the activity of this system and vice versa. Finally, interactions exist between the circadian system, the hypothalamic–pituitary–adrenal axis, and alcohol consumption. Thus, it seems that certain clock genes may control functions of the stress response system and that these interactions are affected by alcohol.

Alcohol abuse and dependence are estimated to affect 1 in 8 adults in the United States and several hundred million people worldwide (Grant et al. 2004). To define at-risk populations and develop better treatments, it is important to further identify the genetic and environmental factors that contribute to alcohol addiction. Recent evidence suggests that the body’s internal system that helps control the daily rhythm of the body’s activities (i.e., the circadian system), the body’s stress response system, and the body’s neurobiology of alcohol are extensively intertwined. This article explores some of these interactions.

## The Circadian System and Alcohol’s Effects on It

The circadian system—or the body’s internal clock—is a naturally present regulatory system that helps the body maintain an approximately 24-hour cycle in biochemical, physiological, or behavioral processes, thereby allowing the organism to anticipate and prepare for regular environmental changes (i.e., the day–night cycle). For example, circadian rhythms maintain not only sleeping and feeding patterns but also physiological processes such as body temperature, brain-wave activity, hormone production, and cell regeneration. The circadian clockwork results from the interaction of specific clock genes, including genes known as *Period* (*Per1, Per2,* and *Per3*), *Clock*, *Bmal1*, and *Cryptochrome* (*Cry1* and *Cry2*), and others.^1^ The activity of these genes is controlled by two tightly coupled transcriptional and translational feedback loops that sustain a near 24-hour periodicity of cellular activity. Expression of these clock genes, in turn, regulates the expression of other clock-controlled genes (Ko and Takahashi 2006).

In both humans and animal models, complex bidirectional relationships seem to exist between alcohol intake or exposure and circadian clock systems. The impact of the circadian system on alcohol use is shown by the fact that both preference for and consumption of alcohol are modulated by time of day, and studies found that genetic interactions link core circadian clock genes with alcohol drinking (Spanagel et al. 2005*a*, *b*). In addition, disruption of the normal circadian rhythm (i.e., circadian desynchronization) seems to increase the use of alcohol, as seen in frequent travelers and rotating-shift workers, possibly because it frequently activates the body’s stress response (i.e., increases the allostatic load^2^) (Rosenwasser et al. 2010; Trinkoff and Storr 1998). At the same time, a strong relationship seems to exist between alcohol drinking and altered circadian functions. For example, alcohol intake can alter the following circadian responses:

- Circadian rhythms in blood pressure, core body temperature, and hormone release in humans (Danel et al. 2009; Devaney et al. 2003; Nakashita et al. 2009);
- Shifts in the normal circadian rhythm (i.e., circadian phase shifting) and in the free-running period^3^ in mice (Prosser et al. 2008; Seggio et al. 2009);
- Return to a normal circadian rhythm after a disruption (i.e., circadian phase resetting) and nocturnal activity patterns in hamsters (Ruby et al. 2009; Seggio et al. 2007); and
- Rhythmicity in the activity of certain brain cells (i.e., proopiomelanocortin [POMC]^4^-producing neurons) in a brain region called the hypothalamus (which is involved in the body’s stress system) in rats (Chen et al. 2004).

Even alcohol exposure before birth can interfere with circadian systems. Thus, prenatal ethanol exposure in rats can alter core body temperature and phase-shifting ability (Sakata-Haga et al. 2006); rhythmic activity of the pituitary gland and the adrenal gland, both of which are part of the body’s stress response system (Taylor et al. 1982); the rhythmic release of the main stress hormone (i.e., corticosterone) (Handa et al. 2006); immune cell rhythms (Arjona et al. 2006); and circadian expression of POMC in the hypothalamus (Chen et al. 2006).

### Why Is the Body’s Circadian System So Vulnerable to Alcohol Toxicity?

One logical explanation for the sensitivity of the circadian system to alcohol suggests that alcohol specifically targets one or more of the genes that regulate circadian functions. Using different experimental designs, researchers have demonstrated that alcohol exposure significantly alters the expression of several core clock genes. For example, in chronic alcohol-drinking rats, circadian expression of *Per1* and *Per2* is significantly disrupted in the hypothalamus (Chen et al. 2006). Likewise, prenatal alcohol exposure alters circadian expression of *Per1* and *Per2* genes in the hypothalamus and in tissues in other parts of the body in rats and mice (Arjona et al. 2006; Chen et al. 2004; Ko and Takahashi 2006). In addition, neonatal alcohol exposure reduces *Cry1* expression in a brain region called the suprachiasmatic nucleus and advances the phase of the *Per2* rhythm in the cerebellum and liver (Farnell et al. 2008). In human studies, the expression of clock genes (*PER*, *CRY,* and *BMAL1*) is reduced in white blood cells of male alcoholic patients (i.e., after chronic alcohol exposure) (Huang et al. 2010), whereas alcohol drinking in healthy males (i.e., acute exposure) increases *BMAL1* expression in these cells (Ando et al. 2010). Finally, variations of the *PER2* gene in which individual DNA building blocks are altered (i.e., single nucleotide polymorphisms [SNPs]) are associated with increased alcohol consumptions in male patients (Spanagel 2005*a*) and adolescent boys (Comasco et al. 2010). These observations suggest that clock genes are targets through which alcohol may alter circadian functions. However, in-depth molecular studies are necessary to elucidate the potential mechanisms by which alcohol directly or indirectly affects clock gene expression and cellular functions.

## Circadian Systems, the Stress Response, and Alcohol Consumption

### The Stress Response System

The circadian system also may be involved in regulating alcohol-drinking behavior by interacting with a hormone system called the hypothalamic–pituitary–adrenal (HPA) axis, which plays a central role in the body’s stress response as well as in reward mechanisms. Stress increases the production of a hormone called corticotrophin-releasing hormone (CRH) in certain cells in a region known as the paraventricular nucleus (PVN) in the hypothalamus. The CRH then is secreted into the blood vessels leading to the pituitary gland, where it interacts with a specific molecule, the CRH receptor1 (CRHR1), on specific cells in the anterior pituitary. In response, these cells begin the synthesis and release of adrenocorticotropic hormone (ACTH) into the circulation. ACTH, in turn, stimulates the release of glucocorticoids (i.e., corticosterone in rats and cortisol in humans) from the outer layer (i.e., cortex) of the adrenal glands that are located on top of the kidneys. The glucocorticoids then act on numerous tissues throughout the organism to coordinate the body’s stress response. However, the CRH/CRHR1 system is found not only in the hypothalamus but also in other areas of the brain and helps mediate the actions of the brain’s central stress response systems.

The CRH–HPA system is controlled by many brain-signaling molecules (i.e., neurotransmitters) and their receptors, including opioid peptides^5^ (e.g., β-endorphin [β-EP]) and their receptors. For example, in rats, the bodies of CRF-producing cells are found in the same locations of the PNV as the fibers of β-EP–releasing cells. In another area of the hypothalamus called the median eminence, a certain type of opioid receptors (i.e., μ-opioid receptors [MOP-r]) is located on the ends of CRH-releasing cells. Agents that stimulate the activity of this receptor (i.e., MOP-r agonists) can inhibit neurotransmitter-stimulated CRF release from the hypothalamus in vitro. Likewise, studies in living organisms found that β-EP infusion decreased CRH release in the blood vessels linking the hypothalamus and the pituitary (Plotsky 1991), and morphine pretreatment prevented stress-induced HPA activation (Zhou et al. 1999). Finally, transplantation of β-EP–producing cells into the PVN suppressed HPA activation under different conditions and normalized stress hyperresponse in fetal alcohol-exposed rats (Boyadjieva et al. 2009). All of these data suggest that endogenous opioids (and, by extension, opiate drugs) have a counterregulatory effect on the stress response.

### Alcohol and the Stress Response

In the central nervous system, β-EP long has been suspected of contributing to the positive reinforcement and motivational properties of several addictive substances. For example, microinjection of this peptide to several regions of the brain’s reward system that involves the neurotransmitter dopamine (i.e., the mesolimbic dopamine system), such as the nucleus accumbens, produced place preference (Bals-Kubik et al. 1993). In addition, several studies have demonstrated that repeated administration of alcohol, cocaine, or heroin significantly attenuated β-EP expression in various limbic areas (Jarjour et al. 2009; Rasmussen et al. 2002; Sweep et al. 1988), supporting the notion that β-EP may contribute significantly in the development of alcohol abuse and dependence.

The stress response system also interacts with these reward pathways. For example, the CRH/CRHR1 system can activate mesolimbic dopaminergic pathways and increase dopamine-mediated signal transmission in various parts of the mesolimbic system, including the nucleus accumbens, amygdala, and medial prefrontal cortex. Furthermore, elevation of plasma corticosterone has been associated with increases in alcohol self-administration (Fahlke et al. 1995). Finally, evidence indicates that corticosterone directly stimulates activity of the mesolimbic dopamine system, subsequently increasing drug-seeking behavior (Piazza et al. 1996). Thus, stress, via activation of the CRH–HPA circuits and/or extrahypothalamic CRH circuits, increases mesolimbic dopamine that, in turn, increases drug seeking in drug-treated animals. The relationship between the stress response and the mesolimbic dopamine system is further supported by findings that an abnormality in POMC-mediated regulation of the HPA axis may lead to excess alcohol drinking under stressful conditions. Finally, consistent with animal studies demonstrating acute and chronic effects of alcohol on the HPA axis (Koob and Bloos 1998), studies in humans have documented HPA axis alterations in both actively drinking and recently abstinent alcoholics (Sinha 2007; Uhart and Wand 2009).

### Circadian Genes, the Stress Response, and Alcohol

Several findings have suggested that interactions exist between the circadian system, the HPA axis, and alcohol-drinking behavior (see the figure). For example, in animal studies, forced-swimming and immobilization stress elevated expression of the murine *Per1* gene in CRH-positive cells of the PVN (Takahashi et al. 2001). On the other hand, stress-related (i.e., cortisol-induced) transcriptional activation of human *PER1* was reduced in a type of human blood cells (i.e., B-lymphoblastoid cells) that carried an altered form of the *PER1* gene (i.e., the rs3027172 genotype), which has been associated with an increased risk of alcoholism (Dong et al. 2011). Moreover, alcohol consumption can decrease *Per2* expression in POMC-producing neurons in the hypothalamus (Chen et al. 2004), and certain mutations in the murine *Per2* gene interfere with alcohol’s stimulatory effect on POMC neurons (Agapito et al. 2010) and alter the rhythmic changes in corticosterone levels in the blood (Yang et al. 2009). Thus, it seems that the *Per1* and *Per2* genes may control functions of CRH- and POMC-producing neurons and that these interactions are affected by alcohol.

It is possible that alcohol-mediated modulation of *Per* genes may play a significant role in modulating HPA axis function, which in turn may lead to an increased propensity to drink alcohol following a stressful event. This view is supported by the recent findings by Dong and colleagues (2011) that the presence of certain *Per1* mutations increased psychosocial stress-induced alcohol drinking in mice, increased alcohol-drinking behavior in human adolescents following psychosocial adversity, and reduced cortisol-induced transcriptional activation of *Per1* in human B-lymphoblastoid cells. Other recent findings, although preliminary, showed that a certain *Per2* mutation increased basal levels of plasma corticosterone and alcohol drinking while preventing stress-induced increases in corticosterone levels and alcohol drinking in mice (Logan et al. 2011). In this context, it is interesting to note that mice carrying mutations in *Per2*, but not *Per1*, display ethanol reinforcement and alcohol-seeking behavior (Spanagel et al. 2005*a*; Zghoul et al. 2007).

## Conclusions

The studies reviewed here suggest an intricate interaction between circadian genes, the body’s stress response, and alcohol consumption. Thus, it seems that particularly the *Per1* and *Per2* genes, which have a distinct influence on the HPA axis, may control stress-induced propensity to alcohol drinking behavior. However, additional research is needed to address this novel concept involving clock genes, stress, and alcohol drinking.

## Figures and Tables

**Figure:**
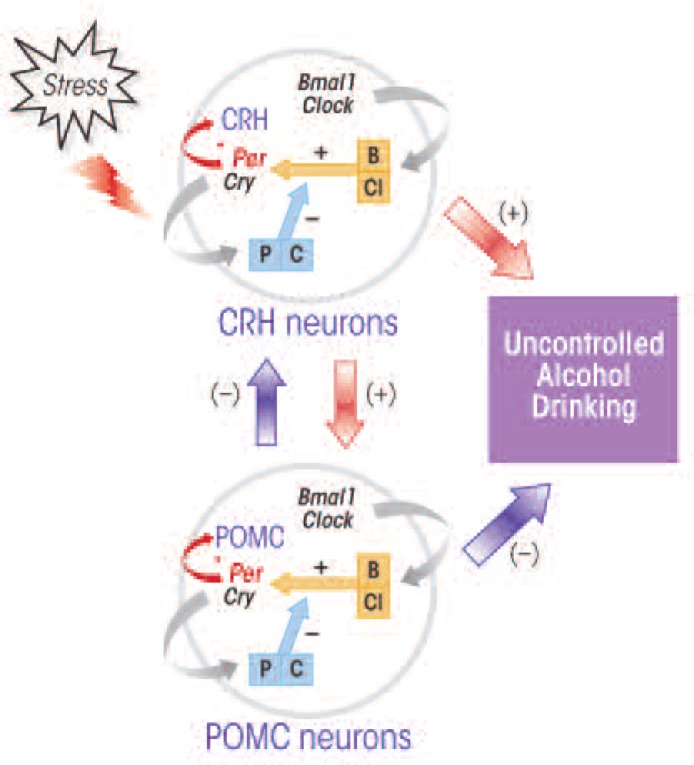
Conceptual framework of how the circadian genes regulating stress-induced excess alcohol drinking. Clock genes (*Per* = P, *Cry* = C, *Bmal1* = B, and *Clock* = Cl) are key components of the circadian mechanism controlling the functions of nerve cells in the hypothalamus and pituitary that produce two molecules important in the body’s stress response—corticotrophin-releasing hormone (CRH) and proopiomelanocortin (POMC). Of these clock genes, *Per* might be a potential target of alcohol (indicated by a * symbol) in CRH and POMC neurons and may control the stress-induced propensity to consume alcohol. NOTE: (+) = stimulatory effect; (–) = inhibitory effect.

## Footnotes

1 By convention, gene names in animals are written in uppercase and lowercase and italicized. Gene names in humans are written in all caps and are italicized, whereas the acronyms for the encoded proteins are all caps but not italicized.

2 The term allostatic load refers to the physiological consequences of chronic exposure to fluctuating or heightened hormonal responses resulting from repeated or chronic stress.

3 Free-running period is a period that is not adjusted or entrained to the 24-hour cycle in nature or to any artificial cycle.

4 POMC is a precursor molecule primarily produced in and secreted by the pituitary gland but also in the hypothalamus. POMC subsequently can be processed in other tissues into numerous different products, which in turn exert specific effects on the organism and play a role in a wide range of physiological processes. One of these products is adrenocorticotropic hormone (ACTH), which is produced in the pituitary gland and is part of the body’s stress response system, the hypothalamic–pituitary–adrenal (HPA) axis.

5 Opioid peptides are short sequences of amino acids (i.e., peptides) that are naturally produced by the body and have effects resembling those of opiate drugs. The three main classes of endogenous opioids are endorphins, enkephalins, and dynorphins. Endorphins also are derived from POMC, which also is the precursor for ACTH.
